# Elevated serum FGF21 is an independent predictor for adverse events in hemodialysis patients from two large centers: a prospective cohort study

**DOI:** 10.1080/0886022X.2023.2256414

**Published:** 2023-09-19

**Authors:** Min Li, Li-qiong Jiang, Meng-yu Zhang, Shu-su Liu, Rejean-Ruiel Regis Sawh, Jing Zheng, Yu Yan, Shi-mei Hou, Ke-qi Lu, Obadele Thorne, Bi-cheng Liu, Qing Qian, Yan-feng Wu, Min Yang, Bin Wang

**Affiliations:** a Department of Nephrology, the Third Affiliated Hospital of Soochow University, Changzhou, China; b Institute of Nephrology, Southeast University Zhongda Hospital, Nanjing, China; c Department of Nephrology, The Affiliated Suzhou Hospital of Nanjing Medical University, Suzhou, China; d Peking University Health Science Centre, Beijing, China; e Department of Nephrology, Georgetown Public Hospital Corporation, Georgetown, Guyana; f Department of Pharmacy, the Third Affiliated Hospital of Soochow University, Changzhou, China; g Department of Neurology, Nanjing Medical University Second Affiliated Hospital, Nanjing, China

**Keywords:** All-cause mortality, fibroblast growth factor 21, hemodialysis, major adverse cardiovascular events, pneumonia

## Abstract

**Introduction:** We explored the relationship and the predictive value of serum fibroblast growth factor 21 (FGF21) with all-cause mortality, major adverse cardiovascular events (MACEs) and pneumonia in hemodialysis (HD) patients.

**Methods:** A total of 388 Chinese HD patients from two HD centers were finally enrolled in this prospective cohort study (registration number: ChiCTR 1900028249) between January 2018 and December 2018. Serum FGF21 was detected. Patients were followed up with a median period of 47 months to record the MACEs and pneumonia until death or 31 December 2022.

**Results:** The incidence of all-cause mortality, MACEs and pneumonia in HD patients were 20.6%, 29.6%, and 34.8%, respectively. The optimal cutoffs for FGF21 to predict all-cause mortality, MACEs and pneumonia were 437.57 pg/mL, 216.99 pg/mL and 112.79 pg/mL. Multivariate Cox regression analyses showed that FGF21, as a categorical variable, was an independent predictor for all-cause mortality, MACEs and pneumonia (HR, 3.357, 95% CI, 2.128–5.295, *p* < 0.001; HR, 1.575, 95% CI, 1.046–2.371, *p* = 0.029; HR, 1.784; 95% CI, 1.124–2.830; *p* = 0.014, respectively). The survival nomogram, MACEs-free survival nomogram and pneumonia-free survival nomogram based on FGF21 constructed for individualized assessment of HD patients had a high C-index with 0.841, 0.706 and 0.734.

**Conclusion:** Higher serum FGF21 is an independent predictor of all-cause mortality, MACEs and pneumonia in HD patients.

## Graphical Abstract

**Figure:**
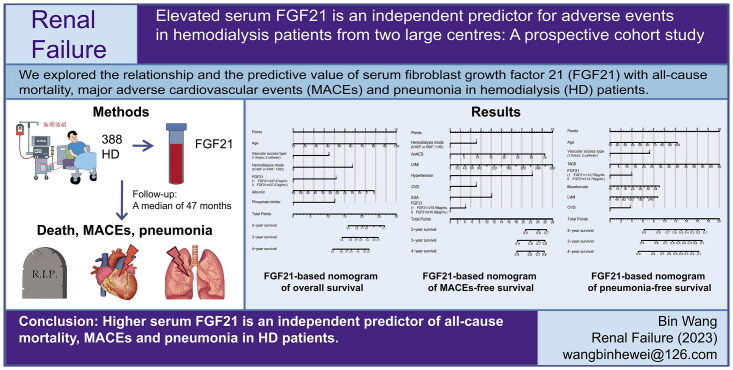

## Introduction

Fibroblast growth factor 21 (FGF21) is mainly produced and secreted by the liver and adipose tissue, which acts as an endocrine-like cytokine in glucose, lipid and energy metabolism [1]. Recent studies have also revealed that circulating FGF21 levels are elevated in several metabolic abnormalities [2,3]. In addition, it also increases progressively as renal function declines [4].

Chronic kidney disease (CKD) dramatically increases the risks of all-cause mortality and comorbidities, especially in patients with end-stage kidney disease (ESKD) [5]. However, the studies on the association of serum FGF21 with all-cause mortality in ESKD patients are inconsistent. While one study has suggested a positive correlation between elevated FGF21 and all-cause mortality [6], another study has shown no correlation between them in HD patients [7]. The incidences of major adverse cardiovascular events (MACEs) and infectious complications, especially pneumonia [8], which are the top two leading causes of death in ESKD patients, are significantly higher than those in the general population [5]. Our previous study showed that circulating FGF21 levels were a potential predictor and promoter of vascular calcification in hemodialysis (HD) patients [9]. However, the studies on the association of circulating FGF21 levels with cardiovascular disease (CVD) or MACEs are inconsistent. While some cross-sectional studies have suggested that elevated FGF21 level was associated with CVD, the development of a MACE in the, future and mortality [10–14], other studies have shown no correlation between them [15–17]. Meanwhile, a possible role for FGF21 in several inflammatory reactions (sepsis, pancreatitis, systemic inflammatory response syndrome and community-acquired pneumonia) and mortality has been demonstrated due to its anti-inflammatory and antioxidative properties [18–21].

Given the effects of FGF21 on oxidative stress, anti-inflammatory and its pleiotropic metabolic actions, FGF21 might be a potentially effective biomarker for the diagnosis, treatment and prognosis of ESKD with cardiovascular or infectious complications. However, up to date, whether FGF21 is correlated with MACEs and pneumonia and the impact of FGF21 on the all-cause mortality in patients with ESKD has not been fully yielded. Therefore, the aim of our study is to explore the relationship between FGF21 and MACEs, pneumonia and all-cause mortality in Chinese ESKD patients.

## Materials and Methods

### Subjects

Between January 2018 and December 2018, a total of 802 HD patients from 2 large HD centers, Nanjing Zhongda Hospital (*n* = 450) and the First People’s Hospital of Changzhou (*n* = 352), China were enrolled in this prospective cohort study. The details of the selection criteria, design, and data collection procedures of the study have been published previously [9]. The exclusion criteria for this study were as follows: age younger than 18 or older than 90 years, HD vintage less than 6 months, acute infections or other acute illnesses, acute heart failure or myocardial infarction, malignancy, parathyroidectomy history, without chest multi-slice computed tomography (MSCT) in half a year, Kt/V for urea < 1.2, nPCR < 1.0 g/(kg·d) or refused to provide informed consent. Consequently, a total of 388 patients who underwent stable regular HD were enrolled in the final analysis. This study was conducted according to the Declaration of Helsinki and Good Clinical Practice Guidelines. The study protocol was approved by the Ethics Committee of the Third Affiliated Hospital of Soochow University, China (registration number 63/2020). Written informed consent was obtained from all recruited subjects or their guardians before enrollment in the study. The registration number of this clinical trial is ChiCTR 1900028249.

### Follow-up and definitions

Patients were followed up after discharge using a standardized protocol that included outpatient follow-up, electronic medical records of rehospitalization, and telephone contacts with patients or family members until 31 December 2022. The primary endpoint was the time and cause of death, while the secondary endpoint was the time to the first episode of MACEs and pneumonia, which were assessed by the senior residents who were blinded to other results. All patients finished the follow-up with complete baseline data.

Diabetes mellitus (DM) was defined as the use of insulin or oral hypoglycemic medications, or meeting the WHO 1999 criteria.

Hypertension (HTN) was defined as a sitting blood pressure ≥ 140/90 mmHg or on antihypertensive medications.

Known CVD at baseline was defined as any history of myocardial infarction, angina, stroke, transient ischemic attack, heart failure, and coronary or peripheral arterial revascularization.

MACEs included cardiac death, heart failure, angina pectoris, myocardial infarction, and stroke.

Cardiac death was defined as death due to any cardiovascular event other than non-cardiac cardiovascular death such as stroke and peripheral vascular disease.

### Anthropometric parameters and biochemical assessment

Details of the clinical and biochemical data are provided in the Supplementary data, Methods.

### Assessment of cardiac function

All patients underwent echocardiography. The measurement of left ventricular mass index (LVMI) is provided in the Supplementary data, Methods.

### Assessment of vascular calcification

Non-enhanced chest computed tomography (CT) was performed on a 64-detector CT scanner (GE Healthcare, USA) within three months of enrollment. As reported previously, we set a CT attenuation > 130 Hounsfield units as the vascular calcification (VC) lesion and measured the thoracic aorta calcification score (TACS), ascending thoracic aorta calcification score (ATACS), aortic arch calcification score (AoACS), and descending thoracic aorta calcification score (DTACS) by Advantage Workstation Volumeshare 5 software (GE Healthcare, USA) [9].

### Statistics

The version 22.0 SPSS software suite and R software 3.3.3 (Package survC1 and PredictABEL) were used for all statistical analyses. For continuous data, the Kolmogorov-Smirnov test was used to evaluate normality. Data were expressed as mean ± standard deviation (SD) for normally distributed variables and median (interquartile range, IQR) for variables with skewed distribution. Qualitative data are presented as the percentage of the study population (%). Skewed distribution data including serum FGF21 levels, FGF23 levels, TACS, ATACS, AoACS, and DTACS were converted into Ln (FGF21), Ln (FGF23), Ln (TACS + 1), Ln (ATACS + 1), Ln (AoACS + 1), and Ln (DTACS + 1) before correlation analysis and Cox regression analysis. Serum FGF21 levels were treated as continuous or divided into two groups according to Youden index in the analysis.

Comparison of clinical values between two groups were performed using chi-square test for categorical variables, and Student’s t test or Mann Whitney *U* test for continuous variables depending on the test for normality. Spearman correlation and Partial correlation analysis was carried out to explore the relationship between circulating FGF21 levels and associated parameters. Multiple logistic regression analysis was performed to identify the independent factors of mortality, MACEs, and pneumonia. Multivariate receiver operating characteristic (ROC) curve analyses were used to determine the corresponding sensitivity and specificity for FGF21 in the identification of mortality and pneumonia. The area under the curve (AUC) and the 95% CI were calculated. The optimal cutoff values of FGF21 were derived from the maximum Youden index (J). Kaplan-Meier curves combined with the log-rank test were used to assess the impact of different FGF21 levels on the cumulative patient overall survival rate, MACEs-free survival rate, and pneumonia-free survival rate. The associations of serum FGF21 level as a continuous variable and a categorical variable respectively with the risk of all-cause mortality, MACEs, and pneumonia over the follow-up period were evaluated using univariable and multivariable Cox proportion hazards regression. The results were presented as hazard ratio (HR) and 95% confidence interval (CI). Factors that were significant in multiple Cox proportional hazards analyses were used for nomograms, whose accuracy was evaluated by the concordance index (C-index) and calibration curves. Two-tailed *p* values < 0.05 were defined as statistically significant.

## Results

### Clinical characteristics

A total of 388 HD patients completed this prospective study. The demographic and biochemical characteristics were summarized in Table 1. Sixty-one patients had chronic glomerulonephritis, 15 had hypertensive nephrosclerosis, 7 had polycystic kidney disease, 3 had IgA nephropathy, 18 had diabetic kidney disease, 4 had ANCA associated systemic vasculitis, 5 had lupus nephritis and 275 had unknown causes.

**Table 1.** Characteristics of subjects and comparison of clinical parameters and laboratory data of HD patients between the high FGF21 group and the low FGF21 group

	All patients	High FGF21 group (FGF21 > 437.57 pg/mL)	Low FGF21 group (FGF21 ≤ 437.57 pg/mL)	*t/ t’/Z/χ2* value	*p* value
Subjects, *n*	388	120	268		
General data					
Age, years	57 ± 16	63.00 (50.50–74.00)	55.00 (44.00–66.00)	−3.645	***<0.001***
Gender, male, %	217 (55.9)	66 (55.0)	153 (57.1)	0.147	0.701
Dialysis vintage, years	2.00 (0.60–5.68)	2.71 (0.50–6.00)	2.00 (0.60–5.56)	−0.261	0.794
Body mass index, kg/m^2^	22.39 (19.95–25.25)	22.17 (19.69–25.30)	22.58 (20.08–25.23)	−0.350	0.726
Body surface area, m^2^	1.66 (1.51–1.78)	1.65 (1.50–1.78)	1.66 (1.51–1.79)	−0.890	0.373
Systolic BP, mm Hg	145 ± 24	147.46 ± 25.84	144.31 ± 23.61	−1.169	0.243
Diastolic BP, mm Hg	82 ± 15	80.89 ± 15.28	82.54 ± 14.32	1.020	0.308
Vascular access type					
Arteriovenous fistula, n, %	366 (94.3)	111 (92.5)	255 (95.1)		
Catheter, n, %	22 (5.7)	9 (7.5)	13 (4.9)	1.088	0.297
Hemodialysis mode					
HD, *n*, %	142 (36.6)	49 (40.8)	93 (34.7)		
HDF, *n*, %	8 (2.1)	4 (3.3)	4 (1.5)		
HD and HDF, *n*, %	238 (61.3)	67 (55.8)	171 (63.8)	3.193	0.223
Blood data					
Haemoglobin, g/L	98 ± 20	96.19 ± 19.51	98.33 ± 20.80	0.941	0.347
Albumin, g/L	36.10 (32.28–39.70)	36.10 (31.30–40.18)	36.25 (32.60–39.63)	−0.824	0.410
Uric acid, mmol/L	366.00 (283.00–464.10)	363.75 (291.13–458.00)	371.60 (282.75-469.33)	−0.485	0.627
Total cholesterol, mmol/L	4.00 (3.35–4.77)	3.75 (3.30–4.49)	4.05 (3.41–4.93)	−1.808	0.071
Triglycerides, mmol/L	1.45 (1.00–2.19)	1.37 (0.97–2.45)	1.53 (1.01–2.13)	−0.291	0.771
Bicarbonate, mmol/L	22.75 ± 3.81	22.17 ± 3.59	23.01 ± 3.88	1.968	0.050
Corrected calcium, mmol/L	2.35 (2.23–2.49)	2.37 (2.26–2.50)	2.34 (2.20–2.49)	−1.128	0.259
Phosphate, mmol/L	1.69 (1.30–2.09)	1.75 (1.31–2.19)	1.68 (1.29–2.04)	−1.424	0.154
Corrected calcium × phosphate, mg^2^/dL^2^	45.28 (36.04–58.53)	48.11 (36.22–64.08)	44.94 (35.94–56.56)	−1.541	0.123
Parathyroid hormone, pg/mL	267.90 (130.08–505.38)	256.65 (143.35–554.90)	272.85 (124.78–472.98)	−0.808	0.936
FGF21, pg/mL	216.99 (96.14–517.07)	702.06 (567.43–1120.53)	140.22 (60.83–238.18)	15.749	***<0.001***
FGF23, pg/mL	5160 (710–13 581)	5462.36 (684.29–15475.69)	5012.71 (721.25–12070.08)	−0.781	0.435
CT data					
TACS, cm^3^	0.77 (0.00–4.28)	2.15 (0.09–9.40)	0.66 (0.00–2.56)	−4.266	***<0.001***
ATACS, cm^3^	0.00 (0.000.02)	0.00 (0.00–0.33)	0.00 (0.00–0.00)	−4.767	***<0.001***
AoACS, cm^3^	0.43 (0.00–-2.11)	0.76 (0.04-5.42)	0.31 (0.00–1.30)	−4.210	***<0.001***
DTACS, cm^3^	0.25 (0.00–1.93)	0.84 (0.00-4.50)	0.14 (0.00–1.07)	−4.401	***<0.001***
Echocardiography					
LVMI, g/m²	124.59 (99.14160.48)	126.21 (103.38-–165.50)	124.14 (93.98–156.45)	−1.570	0.116
Comorbidity					
Diabetes, %	138 (35.6)	47 (39.2)	91 (34.0)	0.982	0.322
Hypertension, %	334 (86.1)	106 (88.3)	229 (85.4)	0.585	0.444
CVD, %	89 (22.9)	32 (26.7)	58 (21.6)	1.175	0.278
Medicine usage					
Vitamin D, %	165 (42.5)	46 (38.3)	122 (45.5)	1.745	0.187
Calcium supplements, %	109 (28.1)	39 (32.5)	73 (27.2)	1.117	0.290
Cinacalcet, %	51 (13.1)	14 (11.7)	37 (13.8)	0.332	0.564
ACEI/ARB, %	100 (25.8)	35 (29.2)	65 (24.3)	1.046	0.306
Phosphate binder, %	309 (79.6)	97 (80.8)	212 (79.1)	0.153	0.696
ESA, %	341 (87.9)	105 (87.5)	236 (88.1)	0.024	0.876
Outcomes					
Death, %	80 (20.6)	42 (35.0)	38 (14.2)	21.954	***<0.001***
MACEs, %	115 (29.6)	42 (35.0)	73 (27.2)	2.394	0.122
Pneumonia, %	135 (34.8)	50 (41.7)	85 (31.7)	3.617	0.057

BP: blood pressure; HD: hemodialysis; HDF: hemodiafiltration; FGF21: fibroblast growth factor 21; FGF23: fibroblast growth factor 23; TACS: thoracic aorta calcification scores; ATACS: ascending thoracic aorta calcification scores; AoACS: aortic arch calcification scores; DTACS: descending thoracic aorta calcification scores; LVMI: left ventricular mass index; CVD: cardiovascular disease; ACEI: angiotensin-converting enzyme inhibitors; ARB: angiotensin receptor blockers; ESA: erythropoiesis-stimulating agents; MACEs: major adverse cardiovascular events. Italic values represented that the comparison of clinical parameters and laboratory data in HD patients between the high FGF21 group and the low FGF21 group was statistically significant, and the *P*-value was less than 0.05.

During a median follow-up of 47 months, the total incidence of all-cause mortality was 20.6% (80 of 388 patients), including 17 patients with cardiac death and 63 patients with noncardiac death. Furthermore, MACEs occurred in 115/388 (29.6%) patients, including 17 cases of cardiac death, 69 of heart failure, 10 of hospitalization due to angina pectoris, seven of myocardial infarction, and 12 of stroke. Additionally, the incidence of pneumonia was 34.8% (135 of 388 patients).

### Comparison of clinical and laboratory characteristics of HD patients with low or high FGF21

After using an optimal serum FGF21 cutoff of 437.57 pg/mL calculated by the Youden index for predicting the occurrence of death, the 388 HD patients in this cohort were divided into high FGF21 group (FGF21 > 437.57 pg/mL) and low FGF21 group (FGF21 ≤ 437.57 pg/mL), respectively. As shown in Table 1, patients with higher serum FGF21 levels were more likely to be older, have significantly higher TACS, ATACS, AoACS, DTACS than those with lower FGF21 levels (*p* < 0.05). Moreover, significantly more patients died in high FGF21 group (*p* < 0.05). There were no additional parameters with significant differences between the two groups.

Correlation analysis showed that the Ln (FGF21) was significantly positively associated with Ln (TACS + 1), Ln (ATACS + 1), Ln (AoACS + 1), and Ln (DTACS + 1) after adjustment for age and systolic blood pressure (SBP) (all *p* < 0.05, Supplementary data, Table S1).

### Serum FGF21 was an independent predictor of all-cause mortality in HD patients

As shown in Table 2, patients in the non-survivor group were older, had lower diastolic blood pressure, total cholesterol, phosphate and higher FGF21, TACS, ATACS, AoACS, DTACS, and were more likely to have hypertension compared to those in the survivor group (*p* < 0.05). Furthermore, in the non-survivor group, more patients tend to choose hemodialysis mode and use catheter while lower proportion of patients were taking phosphate binder and erythropoiesis-stimulating agents (ESA) (*p* < 0.05). The ROC curves showed that the AUC of FGF21 was 0.675 (95% CI, 0.608–0.742, *p* < 0.001) with a sensitivity of 52.5% and a specificity of 74.7% for predicting all-cause mortality in HD patients. The optimal cutoff was 437.57 pg/mL.

**Table 2.** Comparison of clinical parameters and laboratory data of HD patients between the non-survivor group and the survivor group

	Non-survivor	Survivor	*t/ t’/Z/χ2* value	*p* value
Subjects, n	80	308		
General data				
Age, years	68 (57–78.75)	54 (43–66)	−6.644	***<0.001***
Gender, male, %	49 (61.3)	170 (55.2)	0.947	0.376
Dialysis vintage, years	2.38 (0.62–6.13)	1.96 (0.50–5.50)	−0.669	0.503
Body mass index, kg/m^2^	21.72 (19.53–25.47)	22.56 (20.13–25.25)	−0.881	0.379
Body surface area, m^2^	1.67 (1.53–1.79)	1.66 (1.51–1.78)	−0.302	0.762
Systolic BP, mm Hg	144.99 ± 26.01	145.36 ± 23.91	0.122	0.903
Diastolic BP, mm Hg	77.65 ± 13.75	83.19 ± 14.65	3.046	***0.002***
Vascular access type				
Arteriovenous fistula, n, %	69 (86.3)	297 (96.4)		
Catheter, n, %	11 (13.8)	11 (3.6)	10.472	***0.001***
Hemodialysis mode				
HD, n, %	61 (76.3)	81 (26.3)		
HDF, n, %	1 (1.3)	7 (2.3)		
HD and HDF, n, %	18 (22.5)	220 (71.4)	66.593	***<0.001***
Blood data				
Haemoglobin, g/L	97.89 ± 21.51	97.62 ± 20.15	−0.104	0.917
Albumin, g/L	35.80 (31.33–39.73)	36.50 (32.68–39.70)	−1.121	0.262
Uric acid, mmol/L	343.90 (270.18–428.83)	373.00 (290.45–470.00)	−1.44	0.150
Total cholesterol, mmol/L	3.69 (2.97–4.45)	4.04 (3.44–4.90)	−2.875	***0.004***
Triglycerides, mmol/L	1.32 (0.83–2.10)	1.54 (1.05–2.20)	−1.878	0.060
Bicarbonate, mmol/L	23.17 ± 4.25	22.64 ± 3.68	−1.118	0.264
Corrected calcium, mmol/L	2.37 (2.26–2.51)	2.34 (2.21—2.48)	−1.481	0.139
Phosphate, mmol/L	1.57 (1.12–1.95)	1.70 (1.36–2.11)	−2.462	***0.014***
Corrected calcium × phosphate, mg^2^/dL^2^	43.08 (31.75–57.27)	46.11 (37.02–59.06)	−1.731	0.083
Parathyroid hormone, pg/mL	280.90 (128.48–625.43)	266.50 (129.83–477.35)	−0.038	0.970
FGF21, pg/mL	500.62 (172.32–889.43)	189.07 (81.23–445.11)	−4.819	***<0.001***
FGF23, pg/mL	5633.21 (947.20–18150.25)	5020.56 (658.18–12844.31)	−1.471	0.141
CT data				
TACS, cm^3^	4.44 (1.02-–10.94)	0.54 (0.00–2.34)	−6.809	***<0.001***
ATACS, cm^3^	0.00 (0.00–0.75)	0.00 (0.00–0.00)	−5.750	***<0.001***
AoACS, cm^3^	2.40 (0.46-–5.23)	0.22 (0.00–1.19)	−6.856	***<0.001***
DTACS, cm^3^	1.70 (0.17–4.38)	0.10 (0.00–1.11)	−5.808	***<0.001***
Echocardiography				
LVMI, g/m²	127.57 (106.63–163.70)	122.84 (97.60–157.97)	−1.455	0.146
Comorbidity				
Diabetes, %	34 (42.5)	104 (33.8)	2.114	0.146
Hypertension, %	76 (95)	259 (84.1)	6.408	***0.011***
CVD, %	18 (22.5)	72 (23.4)	0.027	0.869
Medicine usage				
Vitamin D, %	27 (33.8)	141 (45.8)	3.743	0.053
Calcium supplements, %	26 (32.5)	86 (27.9)	0.648	0.421
Cinacalcet, %	8 (10.0)	43 (14.0)	0.873	0.350
ACEI/ARB, %	22 (27.5)	78 (25.3)	0.157	0.692
Phosphate binder, %	48 (60.0)	261 (84.7)	23.972	***<0.001***
ESA, %	65 (81.3)	276 (89.6)	4.169	***0.041***

BP: blood pressure; HD: hemodialysis; HDF: hemodiafiltration; FGF21: fibroblast growth factor 21; FGF23: fibroblast growth factor 23; TACS thoracic aorta calcification scores; ATACS: ascending thoracic aorta calcification scores; AoACS: aortic arch calcification scores; DTACS descending thoracic aorta calcification scores; LVMI: left ventricular mass index; CVD: cardiovascular disease; ACEI: angiotensin-converting enzyme inhibitors; ARB: angiotensin receptor blockers; ESA: erythropoiesis-stimulating agents; MACEs: major adverse cardiovascular events.

**Figure 1.**
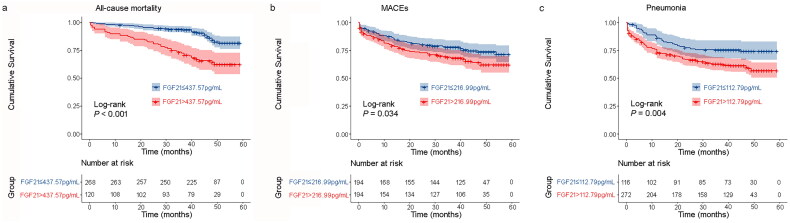
Kaplan–Meier survival analysis for cumulative probability of different clinical outcomes in high and low FGF21 groups. (a) the Kaplan-Meier analysis showed that the overall mortality was significantly higher in the high FGF21 group than in the low FGF21 group (FGF21 ≤ 437.57 pg/mL, log-rank, *p* < 0.001). (b) the Kaplan-Meier analysis showed that the MACEs-free survival rate was higher in the high FGF21 group than in the low FGF21 group (FGF21 ≤ 216.99 pg/mL, log-rank, *p* = 0.034). (c) the Kaplan-Meier analysis showed that the pneumonia-free survival rate was significantly higher in the high FGF21 group than in the low FGF21 group (FGF21 ≤ 112.79 pg/mL, log-rank, *p* = 0.004).

**Figure 2.**
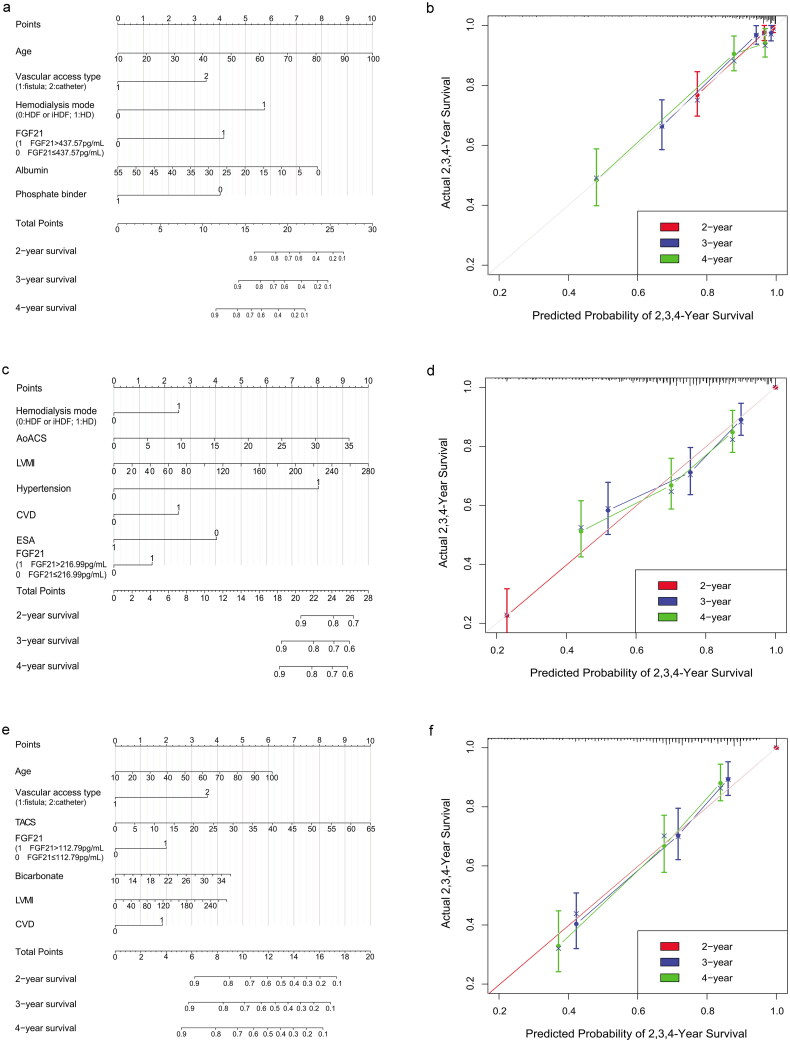
Construction of FGF21-based nomograms in HD patients. (a) FGF21-based nomogram of overall survival. (b) The calibration curves for predicting 2, 3, 4-year survival in HD patients. (c) FGF21-based nomogram of MACEs-free survival. (d) The calibration curves for predicting 2, 3, 4-year MACEs-free survival in HD patients. (e) FGF21-based nomogram of pneumonia-free survival. (d) The calibration curves for predicting 2, 3, 4-year pneumonia-free survival in HD patients. FGF21, fibroblast growth factor 21; HD, hemodialysis; HDF, hemodiafiltration; iHDF: intermittent hemodiafiltration; AoACS, aortic arch calcification scores; LVMI, left ventricular mass index; CVD, cardiovascular disease; ESA, erythropoiesis-stimulating agents; TACS, thoracic aorta calcification scores.

The Kaplan-Meier analysis showed that the all-cause mortality was significantly higher in the high FGF21 group than in the low FGF21 group [35.0% *vs.* 14.2%, log-rank, *p* < 0.001] (Figure 1a). Furthermore, a high circulating FGF21 was confirmed to be an independent predictor of the all-cause mortality in multivariable Cox regression analysis, irrespective of whether serum FGF21 level was expressed as a continuous variable or as a categorical variable (HR, 1.781, 95% CI, 1.443–2.199; HR, 3.357, 95% CI, 2.128-–5.295; all *p* < 0.001) (Supplementary data, Table S2 and Table 3). Based on multivariate Cox regression analysis, we built a nomogram (Figure 2a). FGF21 was utilized as a categorical variable, and the C-index for overall survival prediction was 0.841 (95% CI, 0.795–0.887). The calibration curves for the probability of overall survival demonstrated optimal consistency between the prediction by FGF21-based nomogram and actual observation (Figure 2b). By calculating the score of each factor, the overall survival of the HD patients could be predicted.

**Table 3.** Multivariate Cox regression analyses for all-cause mortality, MACEs and pneumonia prediction in HD patients

Variables	All-cause mortality		Variables	MACEs		Variables	Pneumonia	
	HR (95% CI)	P value		HR (95% CI)	*p* value		HR (95% CI)	*p* value
FGF21 as a continuous variable								
Age, years	1.035 (1.017, 1.053)	*<0.001*	Hemodialysis mode, HD	1.742 (1.168, 2.600)	*0.007*	Age, years	1.020 (1.006, 1.034)	*0.005*
Vascular access type, catheter	3.027 (1.564, 5.860)	*0.001*	AoACS, cm^3^	1.080 (1.034, 1.128)	*0.001*	Vascular access type, catheter	2.673 (1.487, 4.805)	*0.001*
Hemodialysis mode, HD	5.784 (3.367, 9.935)	*<0.001*	LVMI, g/m²	1.007 (1.003, 1.012)	*0.001*	TACS, cm^3^	1.047 (1.026, 1.068)	*<0.001*
Albumin, g/L	0.963 (0.930, 0.998)	*0.040*	Hypertension, yes	3.880 (1.216, 12.384)	*0.022*	LVMI, g/m²	1.005 (1.001, 1.009)	*0.011*
Ln (FGF21)	1.781 (1.443, 2.199)	*<0.001*	CVD, yes	1.657 (1.102, 2.491)	*0.015*	CVD, yes	1.594 (1.083, 2.346)	*0.018*
Phosphate binder, yes	0.320 (0.201, 0.510)	*<0.001*	ESA, yes	0.392 (0.232, 0.663)	*<0.001*			
FGF21 as a categorical variable								
Age, years	1.033 (1.016, 1.051)	*<0.001*	Hemodialysis mode, HD	1.775 (1.190, 2.647)	*0.005*	Age, years	1.020 (1.006, 1.035)	*0.006*
Vascular access type, catheter	2.753 (1.422, 5.332)	*0.003*	AoACS, cm^3^	1.062 (1.013, 1.113)	*0.012*	Vascular access type, catheter	2.658 (1.477, 4.784)	*0.001*
Hemodialysis mode, HD	5.334 (3.104, 9.165)	*<0.001*	LVMI, g/m²	1.007 (1.003, 1.012)	*0.001*	Bicarbonate, mmol/L	1.053 (1.000, 1.108)	*0.050*
Albumin, g/L	0.959 (0.926, 0.993)	*0.020*	Hypertension, yes	4.026 (1.261, 12.850)	*0.019*	TACS, cm^3^	1.040 (1.018, 1.062)	*<0.001*
FGF21 > 437.57 pg/mL	3.357 (2.128, 5.295)	*<0.001*	CVD, yes	1.692 (1.125, 2.545)	*0.012*	LVMI, g/m²	1.004 (1.001, 1.008)	*0.025*
Phosphate binder, yes	0.313 (0.196, 0.502)	*<0.001*	ESA, yes	0.411 (0.243, 0.695)	*0.001*	CVD, yes	1.705 (1.155, 2.519)	*0.007*
			FGF21 > 216.99 pg/mL	1.575 (1.046, 2.371)	*0.029*	FGF21 > 112.79 pg/mL	1.784 (1.124, 2.830)	*0.014*

MACEs: major adverse cardiovascular events; FGF21 fibroblast growth factor 21; HD: hemodialysis; TACS: thoracic aorta calcification scores; AoACS: aortic arch calcification scores; LVMI: left ventricular mass index; CVD: cardiovascular disease; ESA: erythropoiesis-stimulating agents; HR: hazard ratio; CI: confidence interval. Italic values represented that the variable was an independent predictor of all-cause mortality, MACEs or pneumonia. The *P*-value was less than 0.05.

### Serum FGF21 as a categorical variable was independently associated with incident MACEs in HD patients

As shown in Supplementary data, Table S3, participants who developed a MACE tend to be older, have higher TACS, ATACS, AoACS, DTACS, LVMI, and were more likely to have DM, HTN and CVD compared to participants who did not develop a MACE. The proportion of taking angiotensin-converting enzyme inhibitors/angiotensin receptor blockers (ACEI/ARB) drugs was higher while the proportion of taking ESA and undergoing hemodiafiltration was lower in the MACEs group (all *p* < 0.05).

Moreover, according to the median, FGF21 was divided into high group (FGF21 > 216.99 pg/mL) and low group (FGF21 ≤ 216.99 pg/mL). The Kaplan–Meier survival curves showed that the risk of MACEs increased significantly in the high FGF21 group (34.0% *vs.* 25.3%, log-rank, *p* = 0.034) (Figure 1b), which was consistent with the result of the univariate Cox regression analysis (HR, 1.484; 95% CI, 1.025–2.147; *p* = 0.037) (Supplementary data, Table S2). After adjusting for confounding factors, multivariable Cox regression analysis showed that FGF21 was an independent predictor of MACEs when FGF21 levels were assessed as a categorical variable (HR, 1.575; 95% CI, 1.046–2.371; *p* = 0.029) (Table 3).

Based on multivariate Cox regression analysis, we built a FGF21-based nomogram to predict the probability of 2,3,4-year MACEs-free survival in HD patients (Figure 2c). FGF21 was utilized as a categorical variable, and the C-index was 0.706 (95% CI, 0.661–0.751). The calibration curves demonstrated optimal consistency between the prediction by nomogram and actual observation (Figure 2d).

### Serum FGF21 as a categorical variable was an independent predictor of pneumonia in HD patients

The patients in pneumonia group were older, had a longer dialysis vintage, had higher TACS, ATACS, AoACS, DTACS and LVMI, and had a greater serum level of bicarbonate. Moreover, the patients with pneumonia were more likely to use catheter, choose a simple hemodialysis mode, and have a history of hypertension and CVD. Notably, serum FGF21 levels were significantly higher in HD patients with pneumonia than those without. Meanwhile, the significant decrease of diastolic BP, uric acid, phosphate and calcium-phosphorus product in pneumonia group could be observed (Supplementary data, Table S4). ROC curve analyses showed that the optimal cutoff was 112.79 pg/mL for FGF21 to discriminate HD patients with pneumonia from those without, with a sensitivity of 78.5% and specificity of 34.4%. The AUC for FGF21 was 0.564 (95% CI, 0.504–0.624, *p* = 0.037).

The Kaplan-Meier curve showed that compared with the lower FGF21 group, the higher FGF21 group suffered a greater risk of pneumonia (39.0% *vs.* 25.0%, log-rank, *p* = 0.004) (Figure 1c). Similar significant results were obtained in the univariate Cox regression analysis when FGF21 levels were assessed either as a categorical variable or as a ln-transformed variable (HR, 1.160; 95% CI, 1.012–1.329; *p* = 0.033 and HR, 1.811; 95% CI, 1.200–2.731; *p* = 0.005, respectively) (Supplementary data, Table S2). However, after adjusting for other risk factors, the significantly independent correlation between FGF21 and pneumonia event only existed when FGF21 expressed as a categorical variable (HR, 1.784; 95% CI, 1.124–2.830; *p* = 0.014) (Table 3).

We also established a nomogram based on FGF21 to predict the probability of 2, 3, and 4-year pneumonia-free survival in HD patients, with a c-index of 0.734 (95% CI, 0.690–0.778) (Figure 2e). The calibration curves showed a high consistency between the prediction by nomogram and actual observation (Figure 2f).

## Discussion

The current research was the first prospective study to comprehensively analyze the relationship of circulating FGF21 levels with the incidence of MACEs, pneumonia, and all-cause mortality in HD patients from two centers. Our study revealed that serum higher FGF21 was an independent predictor of all-cause mortality, MACEs, and pneumonia in HD patients. Moreover, our study innovatively applied nomogram to validate the individualized predictive value of FGF21 for adverse events in HD patients.

FGF21 is a newly discovered inducible factor which is mainly synthesized and secreted by the liver under physiological conditions. It is also expressed in several other tissues such as adipose tissue, heart, and kidney under stressed or pathological conditions such as injury, systemic inflammation, and kidney impairment [22,23]. The molecular weight of FGF21 is approximately 21 kDa, and it can be cleared through the glomerular filtration barrier. However, most low-flux dialyzers have minimal clearance rates for FGF21 [24]. Furthermore, FGF21 levels increase progressively with decreased renal function [24], reaching 15-fold normal levels in HD patients [25] and 8-fold normal levels in peritoneal dialysis (PD) patients [26]. Stein et al. found that there was a slight increase in circulating FGF21 concentration after hemodialysis, which was statistically significance [25,27]. A recent study also compared the FGF21 concentration at the start and end of HD and found that the plasma FGF21 concentration increased by 29% after hemodialysis, while hemoglobin, hematocrit, and albumin increased by only 8%, 6%, and 10% respectively [27]. Therefore, the increase in plasma FGF21 level exceeded the expected concentration during HD. These results all indicate that FGF21 cannot be cleared through HD, and HD itself may stimulate the release of FGF21 into the circulation. Currently, studies have analyzed the associated factors of FGF21 both in CKD and non-CKD populations. The results on the association of age with serum FGF21 levels are inconsistent. In our study, the concentration of FGF21 was positively associated with age in HD patients, which was in accordance with previous studies in general population and in septic patients [18,28], whereas was in contradiction to a previous study [29]. Besides, FGF21 is positively correlated with hypertension both in elderly outpatients [30] and in youths at risk for metabolic syndrome [31]. Similarly, the link between FGF21 and systolic BP was obtained in our study. Notably, the positive correlation between serum FGF21 levels and TACS in HD patients was also obtained in our study, which was contrary to the result that FGF21 inhibited vascular calcification *in vitro* and animal experiments [32,33], highlighting the need for further studies to explore the exact mechanism of FGF21 elevation on vascular calcification in CKD patients.

In survival analysis, we confirmed that a high FGF21 level was an independent predictor of all-cause mortality in HD patients. Given the complexity of ESKD, a single biomarker may be insufficient to assess its prognosis with high accuracy. Therefore, in the current study, we constructed an effective nomogram combined FGF21 and age, vascular access type, hemodialysis mode, albumin, phosphate binder for individualized assessment of overall survival for each HD patient. It was worth noting that the C-index was high, suggesting that the survival nomogram was a feasible and reliable prognostic tool for individualized prediction of HD patients who need much more vigorous treatment. Similarly, recent studies have yielded the same results in patients with ESKD [6], patients with acute heart failure [34], and patients with diabetes and coronary artery calcification [13]. As to ESKD patients, the difference between our study and the previous Japanese study laid in that our study was a prospective one with more patients enrolled, and the method for calculating the optimal cutoff value of FGF21 was different [6]. In contrast to our results, one study showed a U-shaped association between serum FGF21 levels and all-cause mortality among patients with coronary artery disease [35] and another study showed FGF21 was not an independent predictor of all-cause mortality in HD patients [7]. Except for FGF21, the multivariable Cox regression analysis revealed that phosphate binder usage was a negative predictor of mortality in HD patients. It is well known that hyperphosphatemia is common in ESRD patients and is associated with increased risk of cardiovascular events and death [36]. Therefore, successful treatment of hyperphosphatemia with phosphate binders can decrease poor health outcomes and mortality in ESKD, which was consistent with a previous review [37].

MACEs and pneumonia are the most common complications and leading causes of death in ESKD patients. Nevertheless, the studies on the relationship between baseline serum FGF21 levels and MACEs remain contradictory. Our present study confirmed that FGF21 > 216.99 pg/mL was an independently predictive of MACE occurrence in HD patients, which was in accordance with several previous studies in patients with type 2 diabetes and in patients with cardiovascular diseases [10–13,34,38–40], whereas was inconsistent with other observations [6,15,16]. We hypothesized that the differences in disease spectrum, ethnic background, sample size, and follow-up time may partially be responsible for the discrepancies.

Several studies have reported that FGF21 has anti-inflammatory and anti-oxidative stress properties *via* the nuclear factor (NF)-κB signaling pathways [41,42]. Furthermore, previous studies have found that FGF21 has a protective effect on the kidney by inhibiting inflammation and fibrosis in low-protein diet-induced renal damage and in diabetic nephropathy [43,44]. In addition, FGF21 also plays a key role in hypothalamic [45] and pancreatic inflammation [46]. Conversely, recent clinical studies have demonstrated that FGF21 is positively correlated with serum inflammatory parameters in a state of inflammation. Moreover, the increased serum FGF21 level superior to C-reactive protein and procalcitonin, was significantly correlated with severity and adverse clinical outcome in these patients [18–21, 47]. Our results revealed that the optimal cutoff value of FGF21 for predicting pneumonia was lower than that for predicting all-cause mortality in HD patients. FGF21 > 112.79 pg/mL was independently predictive of incident pneumonia in HD patients. Together with the published data, we hold the opinion that rising FGF21 concentrations may be a compensatory response to resist the progression of pneumonia in HD patients, which heralds an increased risk for pneumonia in these patients.

This prospective study is the first to jointly explore the relationship of serum FGF21 with several clinical outcomes in a cohort of Chinese HD patients from two centers. The particular strengths of the study are as follows: (1) Our study reveals the predictive value of FGF21 in the risk of developing MACEs, pneumonia, and all-cause mortality in HD patients. This can serve as a useful biomarker for clinicians, who are able to use it for risk stratification and potentially guide patient management. (2) The use of a nomogram based on FGF21 to individually assess the adverse events for each HD patient. However, there are also several limitations in this study. First, the sample size is relatively insufficient and the duration of follow-up is short. Second, as no further FGF21 measurements are performed during the follow-up period, the longitudinal analysis of the dynamic changes in FGF21 was unavailable, which may result in selection biases. Third, there was no description of the dialysis dose nor the clearance of solutes by dialysis such as Kt/V, URR and nPCR, and the information about what filters were utilized to perform hemodialysis was also missing, which may affect serum FGF21 concentration and the endpoints, leading to decreased confidence in the results. Forth, the differences in the C index were 0.019, 0 and 0.006 respectively when FGF21 was included or excluded in the all-cause mortality, MACEs and pneumonia prediction models, which suggested that the inclusion of FGF21 had a small impact on the predictability of the models. As a result, our conclusion should be treated with caution. Finally, due to the trial design, we cannot draw firm conclusions about whether serum FGF21 plays a causal role in the outcome among HD patients and we cannot clarify the exact pathophysiological mechanisms of FGF21 in ESKD and its complications. Further multicenter studies with stricter quality control measures, larger sample sizes, and longer follow-up period will be required to evaluate the predictive value of FGF21 and to elucidate whether targeting FGF21 and its signaling pathway will be of therapeutic benefit in HD patients.

## Conclusion

In conclusion, our study revealed that elevated FGF21 was an independent predictive biomarker of the clinical outcomes in HD patients and may be a potential therapeutic target for HD patients.

## Supplementary Material

Supplemental Material

## Data Availability

The data underlying this article will be shared on reasonable request to the corresponding authors.
