# Supplementary material for: Elevated serum FGF21 is an independent predictor for adverse events in hemodialysis patients from two large centers: a prospective cohort study
Source: Ren Fail. 2023 Sep 19;45(2):2256414. doi: 10.1080/0886022X.2023.2256414 (PMC10512844; doi:10.1080/0886022X.2023.2256414)
Supplement: Supplemental Material [file IRNF_A_2256414_SM7509.pdf]

Supplementary Methods:

Anthropometric parameters and biochemical assessment

All participants underwent comprehensive physical examinations and clinical evaluation before the regular HD sessions. The general information, including demographic data (gender, age, dialysis vintage, vascular access type, hemodialysis mode), disease histories of CVD, diabetes mellitus (DM) and hypertension (HTN), and medication at present (vitamin D, calcium supplements, cinacalcet, angiotensin-converting enzyme inhibitors / angiotensin receptor blockers (ACEI / ARB), phosphate binder and erythropoiesis-stimulating agents (ESA)), were systematically gathered using a standardized questionnaire. Height, weight, systolic blood pressure (SBP) and diastolic blood pressure (DBP) were measured by trained nurses using standard protocol and procedures on the same day of drawing bloods. Body mass index (BMI) was calculated as weight in kilograms (kg) divided by height in meters squared. Body surface area (BSA) was calculated using the Stevenson formula as follow: BSA（m^2） = 0.0061 × height (cm) + 0.0128 × weight (kg) - 0.1529.

Fasting blood samples were harvested prior to mid-week dialysis sessions. All of the samples were analyzed immediately for uric acid (UA), calcium (Ca), phosphate, bicarbonate, serum albumin, total cholesterol (TC), triglyceride (TG), hemoglobin (Hb) and intact parathyroid hormone (iPTH) via standard laboratory procedure as described previously. Corrected serum Ca was adjusted to serum albumin level, according to Payne’s formula. In addition, the concentrations of FGF21 and FGF23 were measured from the serum samples stored at -80°C via enzyme-linked immunosorbent assay kits (Neobioscience, China and Joyee Biotechnics, China, respectively) as per the manufacturer’s instructions as previously described. Mean values of three independent measurements were taken for analyses. Intra- and inter-assay coefficients of variation were both less than 10%. Staff measuring serum FGF21

Information Classification: General

and FGF23 were blinded to participant information.

Assessment of cardiac function

All patients underwent echocardiography to obtain left ventricle end-diastolic dimension (LVEDD), interventricular septal end-diastole measurement (IVSd) and posterior wall thickness at end-diastole (PWd) given in centimeters (cm). Left ventricular mass (LVM) was calculated using the following equations: LVM (grams, g) = $0.8 \times \{1.04 \times [(LVEDD + IVSd + PWd)^3 - LVEDD^3]\} + 0.6$. LVM index (LVMI) given in grams per square meters (g/m^2) is the short term for the LVM divided by BSA.

Information Classification: General

Supplementary Tables:

Table S1 Correlation analyses of serum FGF21 levels with other clinical variables in HD patients

Variables	Ln (FGF21)			
	No Adjustment[a]		Adjusted by age, systolic BP[b]	
	R	*P* value	R	*P* value
Age (years)	0.189	<0.001		
Dialysis vintage (years)	-0.012	0.814		
Body mass index (kg/m^2)	-0.020	0.698		
Body surface area (m^2)	-0.044	0.402		
Systolic BP (mmHg)	0.108	0.034		
Diastolic BP (mmHg)	0.007	0.892		
Haemoglobin (g/L)	-0.056	0.275		
Albumin (g/L)	-0.080	0.122		
Uric acid (µmol/L)	0.019	0.706		
Total cholesterol (mmol/L)	-0.065	0.209		
Triglycerides (mmol/L)	0.009	0.863		
Bicarbonate (mmol/L)	-0.079	0.125		
Corrected calcium (mmol/L)	0.099	0.056		
Phosphate (mmol/L)	0.087	0.092		
Parathyroid hormone (pg/mL)	0.081	0.124		
Ln (TACS+1) (cm^3)	0.295	<0.001	0.281	<0.001
Ln (ATACS+1) (cm^3)	0.287	<0.001	0.219	<0.001
Ln (AoACS+1) (cm^3)	0.282	<0.001	0.260	<0.001
Ln (DTACS+1) (cm^3)	0.309	<0.001	0.298	<0.001

Ln (TACS+1), Ln (ATACS+1), Ln (AoACS+1), Ln (DTACS+1) and Ln (FGF21) were used for the analyses.

Information Classification: General

[a] Pearson or Spearman correlation analysis was used.

[b] Partial correlation analysis was used.

BP, blood pressure; FGF21, fibroblast growth factor 21; TACS, thoracic aorta calcification scores; ATACS, ascending thoracic aorta calcification scores; AoACS, aortic arch calcification scores; DTACS, descending thoracic aorta calcification scores.

Information Classification: General

Table S2 Univariate Cox regression analyses of all-cause mortality, MACEs and pneumonia

Variables	All-cause mortality		MACEs		Pneumonia	
	HR (95% CI)	*P* value	HR (95% CI)	*P* value	HR (95% CI)	*P* value
General data						
Age (per years)	1.056 (1.039, 1.073)	***<0.001***	1.025 (1.013, 1.038)	***<0.001***	1.032 (1.020, 1.044)	***<0.001***
Gender (male)	1.270 (0.810, 1.992)	0.297	1.153 (0.795, 1.673)	0.453	0.978 (0.696, 1.373)	0.897
Dialysis vintage (per years)	1.026 (0.984, 1.070)	0.231	1.013 (0.975, 1.052)	0.519	1.043 (1.010, 1.076)	***0.009***
Body mass index (per kg/m^2)	0.981 (0.926, 1.040)	0.515	1.015 (0.968, 1.063)	0.546	0.990 (0.947, 1.035)	0.659
Body surface area (per m^2)	1.031 (0.332, 3.196)	0.958	1.723 (0.688, 4.312)	0.245	0.720 (0.291, 1.782)	0.478
Systolic BP (per mmHg)	1.000 (0.991, 1.009)	0.931	1.005 (0.997, 1.012)	0.214	1.002 (0.995, 1.009)	0.570
Diastolic BP (per mmHg)	0.978 (0.963, 0.993)	***0.004***	0.993 (0.981, 1.006)	0.290	0.988 (0.976, 1.000)	***0.044***
Vascular access type, catheter	3.903 (2.060, 7.391)	***<0.001***	1.681 (0.819, 3.450)	0.157	3.056 (1.752, 5.330)	***<0.001***
Hemodialysis mode, HD	6.432 (3.842, 10.767)	***<0.001***	1.845 (1.279, 2.662)	***0.001***	1.729 (1.232, 2.427)	***0.002***
Blood data						
Haemoglobin (per g/L)	0.999 (0.988, 1.010)	0.879	1.005 (0.996, 1.014)	0.291	1.007 (0.998, 1.015)	0.134
Albumin (per g/L)	0.967 (0.937, 0.999)	***0.043***	0.992 (0.963, 1.021)	0.579	0.999 (0.971, 1.027)	0.922
Uric acid (per μmol/L)	0.999 (0.997, 1.000)	0.111	1.000 (0.998, 1.001)	0.651	0.999 (0.997, 1.000)	0.092
Total cholesterol (per mmol/L)	0.706 (0.564, 0.884)	***0.002***	0.835 (0.700, 0.996)	***0.045***	0.922 (0.791, 1.075)	0.298
Triglycerides (per mmol/L)	0.848 (0.692, 1.039)	0.111	0.944 (0.813, 1.097)	0.454	0.931 (0.808, 1.072)	0.320
Bicarbonate (per mmol/L)	1.034 (0.976, 1.096)	0.258	1.038 (0.989, 1.089)	0.131	1.055 (1.008, 1.103)	***0.021***
Corrected calcium (per mmol/L)	1.970 (0.780, 4.980)	0.152	0.757 (0.336, 1.708)	0.503	1.118 (0.534, 2.340)	0.768
Phosphate (per mmol/L)	0.590 (0.395, 0.882)	***0.010***	0.807 (0.583, 1.118)	0.198	0.686 (0.503, 0.935)	***0.017***
Corrected calcium×phosphate, mg^2/dL^2	0.992 (0.981, 1.004)	0.210	0.994 (0.984, 1.004)	0.242	0.991 (0.982, 1.000)	0.053
Parathyroid hormone (per pg/mL)	1.000 (0.999, 1.001)	0.951	1.000 (0.999, 1.000)	0.122	1.000 (0.999, 1.000)	0.332

Information Classification: General

Ln (FGF21)	1.693 (1.370, 2.093)	***<0.001***	1.176 (1.015, 1.363)	***0.031***	1.160 (1.012, 1.329)	***0.033***
FGF21>437.57 pg/mL[a]	2.968 (1.912, 4.606)	***<0.001***	-	-	-	-
FGF21> 112.79 pg/mL[b]	-	-	-	-	1.811 (1.200, 2.731)	***0.005***
FGF21>216.99 pg/mL[c]	-	-	1.484 (1.025, 2.147)	***0.037***	-	-
Ln (FGF23)	1.075 (0.945, 1.223)	0.270	1.026 (0.927, 1.137)	0.616	1.076 (0.977, 1.184)	0.135
CT data						
TACS (per cm^3)	1.043 (1.029, 1.058)	***<0.001***	1.029 (1.013, 1.045)	***<0.001***	1.037 (1.023, 1.050)	***<0.001***
ATACS (per cm^3)	1.347 (1.215, 1.495)	***<0.001***	1.164 (1.033, 1.311)	***0.013***	1.177 (1.061, 1.305)	***0.002***
AoACS (per cm^3)	1.097 (1.067, 1.129)	***<0.001***	1.062 (1.028, 1.097)	***<0.001***	1.074 (1.044, 1.104)	***<0.001***
DTACS (per cm^3)	1.071 (1.038, 1.106)	***<0.001***	1.053 (1.020, 1.087)	***0.001***	1.077 (1.048, 1.107)	***<0.001***
Echocardiography						
LVMI (per g/m^2)	1.004 (1.000, 1.008)	0.076	1.007 (1.004, 1.011)	***<0.001***	1.006 (1.003, 1.009)	***0.001***
Comorbidity						
Diabetes (yes)	1.400 (0.898, 2.181)	0.137	1.520 (1.051, 2.198)	***0.026***	1.084 (0.764, 1.537)	0.652
Hypertension (yes)	2.966 (1.084, 8.111)	***0.034***	7.035 (2.234, 22.148)	***0.001***	2.581 (1.312, 5.076)	***0.006***
CVD (yes)	0.922 (0.545, 1.560)	0.762	2.399 (1.643, 3.504)	***<0.001***	2.077 (1.457, 2.961)	***<0.001***
Medicine usage						
Vitamin D (yes)	0.663 (0.417, 1.055)	0.083	0.810 (0.557, 1.178)	0.270	0.769 (0.543, 1.090)	0.140
Calcium supplements (yes)	1.238 (0.776, 1.978)	0.370	0.863 (0.569, 1.309)	0.488	0.931 (0.638, 1.359)	0.711
Cinacalcet (yes)	0.691 (0.333, 1.434)	0.321	0.777 (0.436, 1.384)	0.391	0.729 (0.426, 1.247)	0.249
ACEI/ARB (yes)	0.993 (0.607, 1.623)	0.977	1.541 (1.047, 2.268)	***0.028***	1.127 (0.775, 1.641)	0.531
Phosphate binder, yes	0.316 (0.201, 0.496)	***<0.001***	0.658 (0.431, 1.003)	0.052	0.749 (0.504, 1.115)	0.155
ESA, yes	0.524 (0.298, 0.919)	***0.024***	0.570 (0.352, 0.923)	***0.022***	0.796 (0.490 1.294)	0.358

Data are expressed as HR (95% CI) in terms of per SD or median increase in continuous variables

a: After calculation of the Youden index, the cut-off value of a FGF21 for predicting the occurrence of death was 437.57 pg/mL.

Information Classification: General

b: After calculation of the Youden index, the cut-off value of a FGF21 for predicting the occurrence of pneumonia was 112.79 pg/mL.

c: The median of FGF21 was 216.99 pg/mL.

BP, blood pressure; HD, hemodialysis; FGF21, fibroblast growth factor 21; FGF23, fibroblast growth factor 23; TACS, thoracic aorta calcification scores; ATACS, ascending thoracic aorta calcification scores; AoACS, aortic arch calcification scores; DTACS, descending thoracic aorta calcification scores; LVMI, left ventricular mass index; CVD, cardiovascular disease; ACEI, angiotensin-converting enzyme inhibitors; ARB, angiotensin receptor blockers; ESA, erythropoiesis-stimulating agents; MACEs, major adverse cardiovascular events; HR, hazard ratio; CI, confidence interval.

Information Classification: General

Table S3 Comparison of clinical parameters and laboratory data of HD patients between the MACEs group and the non- MACEs group

	MACEs (+)	MACEs (-)	*t*/ *t'*/*Z*/χ2 value	*P* value
Subjects, n	115	273		
General data				
Age, years	63.00 (53.00-70.00)	55.00 (43.00-66.00)	-3.671	***<0.001***
Gender, male, %	68 (59.1)	151 (55.3)	0.480	0.488
Dialysis vintage, years	2.08 (0.75-6.00)	1.83 (0.50-5.36)	-0.675	0.500
Body mass index, kg/m^2	22.95 (20.23-26.03)	22.32 (19.83-25.19)	-0.705	0.481
Body surface area, m^2	1.68 (1.53-1.81)	1.65 (1.50-1.78)	-1.392	0.164
Systolic BP, mm Hg	147.86±21.56	144.20±25.36	-1.343	0.180
Diastolic BP, mm Hg	81.14±14.16	82.41±14.82	0.770	0.442
Vascular access type				
Arteriovenous fistula, n, %	107 (93.0)	259 (94.9)		
Catheter, n, %	8 (7.0)	14 (5.1)	0.506	0.477
Hemodialysis mode				
HD, n, %	55 (47.8)	87 (31.9)		
HDF, n, %	0 (0.0)	8 (2.9)		
HD and HDF, n, %	60 (52.2)	178 (65.2)	10.973	***0.004***
Blood data				
Haemoglobin, g/L	99.89±19.37	96.75±20.80	-1.371	0.171
Albumin, g/L	35.60 (31.78-40.03)	36.50 (32.50-39.60)	-0.601	0.548
Uric acid, mmol/L	374.70 (295.25-452.37)	360.75 (282.48-467.83)	-0.112	0.911
Total cholesterol, mmol/L	3.88 (3.23-4.62)	4.04 (3.44-4.85)	-1.484	0.138
Triglycerides, mmol/L	1.40 (0.94-1.97)	1.51 (1.02-2.30)	-1.424	0.154
Bicarbonate, mmol/L	23.21±3.79	22.56±3.80	-1.532	0.126
Corrected calcium, mmol/L	2.34 (2.20-2.47)	2.35 (2.24-2.50)	-0.703	0.482

Information Classification: General

Phosphate, mmol/L	1.66 (1.19-2.11)	1.69 (1.34-2.09)	-1.129	0.259
Corrected calcium×phosphate, mg^2/dL^2	43.85 (32.28-59.00)	45.99 (37.07-58.47)	-1.248	0.212
Parathyroid hormone, pg/mL	230.40 (116.40-423.60)	274.30 (141.25-529.95)	-1.765	0.078
FGF21, pg/mL	250.84 (109.50-582.88)	202.00 (90.19-480.14)	-1.710	0.087
FGF23, pg/mL	4994.86 (842.78-13290.83)	5306.67 (685.14-13658.50)	-0.379	0.705
CT data				
TACS, cm^3	1.76 (0.31-5.42)	0.54 (0.00-3.20)	-4.058	***<0.001***
ATACS, cm^3	0.00 (0.00-0.12)	0.00 (0.00-0.00)	-2.948	***0.003***
AoACS, cm^3	0.81 (0.21-3.16)	0.20 (0.00-1.33)	-4.458	***<0.001***
DTACS, cm^3	0.55 (0.01-2.56)	0.10 (0.00-1.49)	-3.36	***0.001***
Echocardiography				
LVMI, g/m^2	139.75 (112.55-172.23)	117.60 (93.70-153.71)	-3.763	***<0.001***
Comorbidity				
Diabetes, %	50 (43.5)	88 (32.2)	4.464	***0.035***
Hypertension, %	112 (97.4)	223 (81.7)	16.925	***<0.001***
CVD, %	43 (37.4)	47 (17.2)	18.487	***<0.001***
Medicine usage				
Vitamin D, %	45 (39.1)	123 (45.1)	1.157	0.282
Calcium supplements, %	30 (26.1)	82 (30.0)	0.615	0.433
Cinacalcet, %	13 (11.3)	38 (13.9)	0.485	0.486
ACEI/ARB, %	39 (33.9)	61 (22.3)	5.661	***0.017***
Phosphate binder, %	86 (74.8)	223 (81.7)	2.377	0.123
ESA, %	95 (82.6)	246 (90.1)	4.277	***0.039***

BP, blood pressure; HD, hemodialysis; HDF, hemodiafiltration; FGF21, fibroblast growth factor 21; FGF23, fibroblast growth factor 23; TACS, thoracic

Information Classification: General

aorta calcification scores; ATACS, ascending thoracic aorta calcification scores; AoACS, aortic arch calcification scores; DTACS, descending thoracic aorta calcification scores; LVMI, left ventricular mass index; CVD, cardiovascular disease; ACEI, angiotensin-converting enzyme inhibitors; ARB, angiotensin receptor blockers; ESA, erythropoiesis-stimulating agents; MACEs, major adverse cardiovascular events.

Information Classification: General

Table S4 Comparison of clinical parameters and laboratory data of HD patients between the pneumonia group and the non-pneumonia group

	Pneumonia (+)	Pneumonia (-)	*t/ t'/Z/χ2* value	*P* value
Subjects, n	135	253		
General data				
Age, years	64.00 (54.00-72.00)	54.00 (42.00-66.00)	-5.100	***<0.001***
Gender, male, %	75 (55.6)	144 (56.9)	0.066	0.797
Dialysis vintage, years	2.54 (1.00-6.42)	1.54 (0.50-5.17)	-2.394	***0.017***
Body mass index, kg/m^2	22.81 (19.92-25.19)	22.32 (19.96 -25.28)	-0.027	0.979
Body surface area, m^2	1.66 (1.50-1.78)	1.66 (1.51-1.79)	-0.420	0.675
Systolic BP, mm Hg	146.39±23.64	144.7±24.71	-0.648	0.517
Diastolic BP, mm Hg	80.00±14.98	83.10±14.35	1.980	***0.048***
Vascular access type				
Arteriovenous fistula, n, %	121 (89.6)	245 (96.8)		
Catheter, n, %	14 (10.4)	8 (3.2)	8.552	***0.003***
Hemodialysis mode				
HD, n, %	62 (45.9)	80 (31.6)		
HDF, n, %	1 (0.7)	7 (2.8)		
HD and HDF, n, %	72 (53.3)	166 (65.6)	8.494	***0.012***
Blood data				
Haemoglobin, g/L	100.22±20.14	96.33±20.47	-1.769	0.078
Albumin, g/L	35.70 (31.70-40.20)	36.50 (32.50-39.60)	-0.214	0.831
Uric acid, mmol/L	348.80 (271.00-429.90)	373.00 (293.00-477.00)	-2.030	***0.042***
Total cholesterol, mmol/L	3.99 (3.29-4.86)	4.00 (3.43-4.75)	-0.269	0.788
Triglycerides, mmol/L	1.39 (0.90-2.20)	1.54 (1.04-2.18)	-1.194	0.232

Information Classification: General

Bicarbonate, mmol/L	23.20 (20.70-25.60)	22.00 (19.90-25.00)	-2.400	***0.016***
Corrected calcium, mmol/L	2.36 (2.23-2.50)	2.35 (2.23-2.49)	-0.156	0.876
Phosphate, mmol/L	1.60 (1.14-2.02)	1.71 (1.41-2.10)	-2.478	***0.013***
Corrected calcium×phosphate, mg^2/dL^2	40.73 (31.16-58.38)	46.64 (38.25-58.56)	-2.409	***0.016***
Parathyroid hormone, pg/mL	255.00 (118.80-489.00)	274.00 (141.25-507.95)	-0.848	0.396
FGF21, pg/mL	266.29 (122.00-627.00)	199.86 (83.31-473.21)	-2.082	***0.037***
FGF23, pg/mL	6072.22 (848.33-16103.89)	4842.00 (661.36-12475.28)	-1.428	0.153
CT data				
TACS, cm^3	2.29 (0.41-8.11)	0.44 (0.00-2.15)	-5.656	***<0.001***
ATACS, cm^3	0.00 (0.00-0.23)	0.00 (0.00-0.00)	-5.263	***<0.001***
AoACS, cm^3	1.26 (0.16-3.77)	0.20 (0.00-1.05)	-5.468	***<0.001***
DTACS, cm^3	1.22 (0.01-3.00)	0.09 (0.00-0.83)	-5.204	***<0.001***
Echocardiography				
LVMI, g/m^2	141.00 (112.23-166.64)	116.51 (96.29-152.67)	-3.516	***<0.001***
Comorbidity				
Diabetes, %	50 (37.0)	88 (34.8)	0.195	0.659
Hypertension, %	126 (93.3)	209 (82.6)	8.585	***0.003***
CVD, %	47 (34.8)	43 (17.0)	15.689	***<0.001***
Medicine usage				
Vitamin D, %	51 (37.8)	117 (46.2)	2.571	0.109
Calcium supplements, %	37 (27.4)	75 (29.6)	0.215	0.643
Cinacalcet, %	15 (11.1)	36 (14.2)	0.750	0.387
ACEI/ARB, %	38 (28.1)	62 (24.5)	0.610	0.435
Phosphate binder, %	103 (76.3)	206 (81.4)	1.427	0.232
ESA, %	116 (85.9)	225 (88.9)	0.748	0.387

BP, blood pressure; HD, hemodialysis; HDF, hemodiafiltration; FGF21, fibroblast growth factor 21; FGF23, fibroblast growth factor

Information Classification: General

23; TACS, thoracic aorta calcification scores; ATACS, ascending thoracic aorta calcification scores; AoACS, aortic arch calcification scores; DTACS, descending thoracic aorta calcification scores; LVMI, left ventricular mass index; CVD, cardiovascular disease; ACEI, angiotensin-converting enzyme inhibitors; ARB, angiotensin receptor blockers; ESA, erythropoiesis-stimulating agents.

Information Classification: General
